# Is There a Relative Age Effect among the Most Successful Track and Field Athletes?

**DOI:** 10.5114/jhk/174497

**Published:** 2023-11-28

**Authors:** Eduard Bezuglov, Nadezhda Semeniuk, Maria Shoshorina, Evgeny Savin, Zbigniew Waśkiewicz, Anton Emanov, Georgiy Malyakin, Danila Telyshev, Ryland Morgans

**Affiliations:** 1 Department of Sports Medicine and Medical Rehabilitation, Sechenov First Moscow State Medical University, Moscow, Russia.; 2 High Performance Sports Laboratory, Sechenov First Moscow State Medical University, Moscow, Russia.; 3 Academy of Talents, Moscow, Russia.; 4 Institute of Sport Science, Jerzy Kukuczka Academy of Physical Education, Katowice, Poland.

**Keywords:** youth athletes, athletic events, senior athletes, RAE

## Abstract

The prevalence of the Relative Age Effect (RAE) was studied among medalists from the World Athletics Championships at U18, U20 and Senior age groups and from the Olympic Games from 2000 to 2022. The specific events examined were the 100, 200, 400, 800, 1500, and 3000/5000 m, the long jump, the triple jump, the high jump and the pole vault. Dates of birth from 1,858 outdoor track and field athletes were analysed and further divided into four groups according to the quartile of birth. The RAE was found to be widespread among athletes of both sexes in U18 and U20 age groups in all examined disciplines. There was no difference between the most successful U18 and U20 athletes (p = 0.52). Among senior athletes of both sexes, this effect was not detected and the number of “late-born” athletes in this age group was higher than athletes born in the first three quarters. The prevalence of the RAE across the four groups of events was found in U18 and U20 age groups. Additionally, within each age group, the difference among events was statistically significant. In most successful track and field athletes, the RAE is only significant in U18 and U20 age groups. In senior athletes, the number of “late-born” athletes is significant while RAE disappears. These data may be considered when assessing the athletic potential of an individual athlete.

## Introduction

Over the last few decades, one of the frequently researched topics has been the relative age effect (RAE) across different sports. Interest in this topic has grown due to the negative impact of the RAE on equal competition opportunities for children and young people in the most competitive sporting organisations (Cobley et al., 2009).

The RAE now refers to the over-representation of athletes born closer to the date used to divide athletes into specific age groups (Wattie et al., 2008). The most common date is the 1^st^ of January to the 31^st^ of December for clustering (Pedersen et al., 2022), however, in England and Australia, it is the 1^st^ of September to the 31^st^ of August. In this method, young athletes with a relatively higher chronological age benefit from being more physically developed and from the greater opportunities to acquire various skills and abilities over their relatively younger peers (Hill et al., 2015; Wattie et al., 2015). Although higher chronological age does not necessarily imply higher biological maturity status, as these constructs are uncorrelated, it does seem clear that “early-born” athletes are more likely to have higher biological maturity status than “late-born” athletes. Thus, it clearly appears that there are early-, on-time and late-maturing children, and the degree of their physical and cognitive development is comparable with their maturity status (Amanda Johnson et al., 2017).

The RAE is widespread during adolescence in the most competitive sports and its spread is linked to various socio-cultural factors, including parents, coaches and agents of athletes, as well as athletes themselves. These factors play an important role in the development of the RAE in addition to biological aspects (Hancock et al., 2013). The RAE is most pronounced in the most popular sports such as soccer, hockey as well as track and field. These sports are often regarded as early-specialisation, where the number of athletes born in the first quarter ("early-born") may be significantly greater than those born in the fourth quarter ("late-born") (Garcia-Rubio et al., 2022; Gil et al., 2021; Gundersen et al., 2022; Perez-Gonzalez et al., 2021). Nevertheless, it may be considered that under-represented “late-born” athletes are more likely to reach the professional level and are as successful as “early-born” athletes at an elite level (Fumarco et al., 2017; Morganti et al., 2022; Romann et al., 2021). Moreover, the RAE is even present in less popular team and individual sports, such as handball and cross-country skiing (Roaas et al., 2022; de la Rubia et al., 2021).

The negative impact of the RAE on primary selection and the selection process in the most competitive sporting organisations can be considered proven, although measures to reduce the impact are still not systematically applied in practice (Helsen et al., 2012; Lemoyne et al., 2023; Ruscello et al., 2023).

There are few studies examining this effect among most successful soccer and hockey players. There is a recent study by Kirkeberg et al. (2022) that evaluated the prevalence of the RAE among the best national level track and field athletes at different age groups across all disciplines. It was demonstrated that the RAE decreased with age, but the effect was still present in the senior age group in both male and female athletes (Kirkeberg et al., 2022). Another large study involving French track and field athletes of different ages revealed the presence of the RAE, which increased with the level of competitiveness (Difernand et al., 2023).

Brustio et al. (2021) showed the prevalence of the RAE among young sprinters of both sexes, including elite sprinters (top 100 and top 50 in the world). Rueger et al. (2022) demonstrated the widespread of this effect among young Swiss female jumpers. According to Boccia et al. (2021) throwers of both genders considered elite in the junior category showed a large RAE. Interestingly, male throwers who reached the elite level in the senior category also showed an appreciable RAE (Boccia et al., 2021).

However, there are still no studies analysing the prevalence of the RAE among the most successful track and field athletes (World Championships and Olympic medalists) across varying age groups.

Thus, the aim of this study was to investigate the prevalence of the RAE among the most successful track and field athletes in different age groups. The study tested the hypothesis that the prevalence of the RAE was most present among young athletes and started declining in the senior group, but there were still more “early-born” athletes than “late-born” among them.

## Methods

### Participants

The present study included most successful track and field athletes from different age categories and different events. Medalists in international championships were defined as most successful athletes.

A total of 1,858 athletes' birth dates were analysed and sub-divided into four age groups according to a birth quartile:

- first quarter (Q1) January–March, “early-born”,
- second quarter (Q2) April–June,
- third quarter (Q3) July–September,
- fourth quarter (Q4) October–December, “late-born”.

The study was performed in accordance with the Declaration of Helsinki and approved by the Ethics Committee of the Sechenov First Moscow State Medical University (approval code: N 22-11; approval date: 09 December 2021). The data were obtained from open access sources, therefore no informed consent for study participation was necessary.

### Measures

The number of athletes born in each quartile of all age groups and events was considered. These data were compared between and within both sexes, events and age groups.

### Design and Procedures

The prevalence of the RAE was examined among medalists in world championships of U18 (9 championships), U20 (9 championships) and Senior (11 championships and 6 Olympic Games) age groups for outdoor athletes only from 2000 to 2022. A cross-sectional observational study design was applied (level of evidence 3). The events were selected as the rules were standardised across all age groups. All events were further divided into four groups: Group 1 (100 m, 200 m and 400 m), Group 2 (800 m, 1500 m and 3000/5000 m), Group 3 (long jump and triple jump), and Group 4 (pole vault and high jump). The worldathletics.org website was used for a manual search.

One athlete could only enter each age group once, i.e., if an athlete won the World Senior Championships several times, only one of the results in the particular age group was included in analysis. Moreover, if a U18 or U20 athlete won a World Championship in the Senior group or an Olympic Games, the result was included in the age group at the time of the performance.

### Statistical Analysis

The data were analysed using the statistical package IBM SPSS v. 26.0, Armonk, New York; USA. Frequency analysis was used to describe the RAE. A Chi-square with an arbitrary number of cells was used to compare the prevalence of the RAE among the analysed groups (by age (U18, U20, Senior), gender and discipline (Group 1, 2, 3 and 4)). For 2x2 tables, odds ratios and confidence intervals were calculated for statistically significant results. Results were considered significant at *p* < 0.05.

## Results

**Figure 1:**
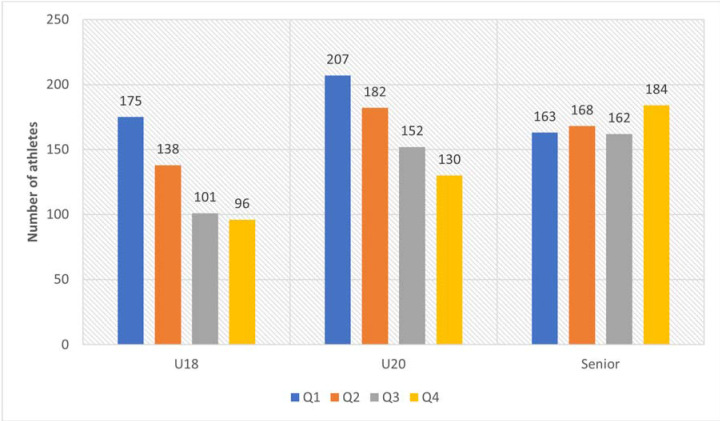
Distribution of birth dates by quarter of the most successful track and field athletes of different age groups.

The prevalence of the RAE was only found in the U18 and U20 age groups. Among the most successful senior athletes, this effect was not reported, although the number of ”late-born” athletes in this age group was higher than athletes born in the first three quarters (Figure 1).

When comparing the RAE among athletes of different age groups, no difference was found between the most successful U18 and U20 athletes (*p* = 0.52).

With regard to the U18 and Senior age groups, a significant difference in the severity of the RAE was observed (*p* < 0.001). This was associated with a significantly higher number of ”early-born” athletes (Q1) (OR = 1.65 (1.28–2.12)) and a significantly lower number of ”late-born” athletes (Q4) (OR = 0.62 (0.47–0.82)) in the U18 compared with the Senior age group. When comparing the U20 and the Senior age groups, a difference was found in the prevalence of the RAE (*p* = 0.002). A pairwise comparison of these groups showed that the U20 age group included significantly more ”early-born” athletes (Q1) (OR = 1.41 (1.11–1.79)) and significantly fewer ”late-born” athletes (Q4) (OR = 0.64 (0.50–0.83)). However, there were no sex differences within each age group (Table 1).

**Table 1:** Comparison of the RAE prevalence among the most successful track and field athletes of both sexes in different age groups.

Age group	Gender	Q1 (n/%)	Q2 (n/%)	Q3 (n/%)	Q4 (n/%)	*p*	Odds Ratio
**U18**	Female	90 (35.6)	62 (24.5)	54 (21.3)	47 (18.6)	0.56	Q1: *p* > 0.05, OR 0.89 (0.62–1.28)
	Male	85 (33.1)	76 (29.5)	47 (18.3)	49 (19.1)		Q4: *p* > 0.05, OR 1.07 (0.68–1.66)
**U20**	Female	107 (32.0)	83 (24.9)	76 (22.8)	68 (20.3)	0.59	Q1: *p* > 0.05, OR 1.12 (0.8–1.55)
	Male	100 (29.7)	99 (29.4)	76 (22.5)	62 (18.4)		Q4: *p* > 0.05, OR 1.13 (0.77–1.66)
**Senior**	Female	76 (23.9)	82 (25.8)	72 (22.6)	88 (27.7)	0.87	Q1: *p* > 0.05, OR 0.98 (0.69–1.4)
	Male	87 (24.2)	86 (24.0)	90 (25.1)	96 (26.7)		Q4: *p* > 0.05, OR 1.05 (0.75–1.47)

**Figure 2:**
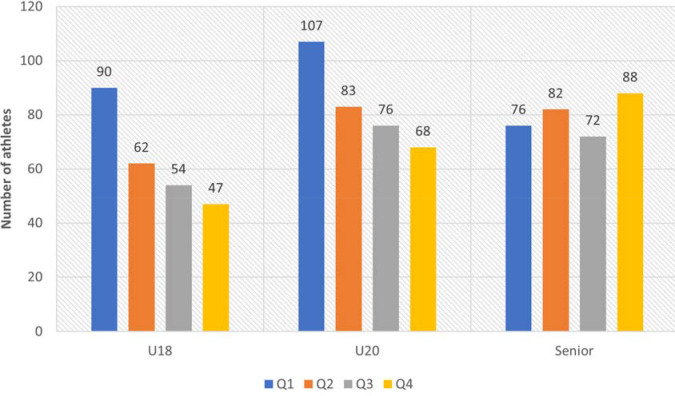
RAE prevalence among the most successful female track and field athletes.

Analysis of the RAE among most successful female track and field athletes demonstrated a statistically significant difference in its prevalence across all age groups (*p* < 0.05) (Figure 2). In a pairwise comparison between U18 and U20 age groups, there was no difference (*p* = 0.82), yet a statistically significant difference (*p* = 0.009) was found between U18 and Senior age groups, which was expressed by the fact that there were more ”early-born” female athletes at U18 (Q1) (OR 1.75 (1.21–2.51)) and less ”late-born” (Q4) (OR 0.49 (0.33–0.72)). However, no statistically significant difference in the RAE prevalence was found among athletes from the U20 and Senior age groups (*p* = 0.057).

**Figure 3:**
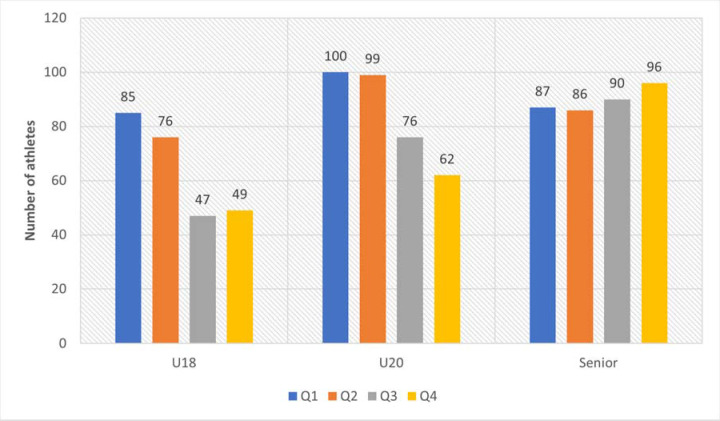
RAE prevalence among the most successful male track and field athletes.

When analysing the RAE among most successful male track and field athletes, a statistically significant difference in its prevalence across age groups was demonstrated (*p* < 0.05) (Figure 3). In a pairwise comparison of the RAE prevalence among male track and field athletes in the U18 and U20 age groups, no statistically significant difference was found (*p* = 0.60). When comparing the prevalence of the RAE among male track and field athletes in U18 and Senior groups as well as U20 and Senior age groups, a statistically significant difference was found in both cases (*p* = 0.006 and *p* = 0.023, respectively) and it was highlighted by both the U18 and U20 age groups having more ”early-born” track and field athletes (Q1: OR 1.55 (1.08–2.2) – U18, 1.32 (0.94–1.85) – U20) and less ”late-born” (Q4: OR 0.63 (0.42–0.93) – U18, 0.69 (0.49–0.99) – U20) than the senior age group (*p* = 0.023).

The prevalence of the RAE across the four groups of events was found in U18 and U20 age groups. There were no statistically significant gender differences in the RAE in either group. Additionally, within each age group, the difference among events was statistically significant (Table 2).

**Table 2:** Comparison of the RAE prevalence among the most successful track and field athletes of both sexes of different age groups competing in different events.

Age Group	Event	Gender	Q1 (n/%)	Q2 (n/%)	Q3 (n/%)	Q4 (n/%)	*p*
**U18**	1	Female	20 (29.0)	16 (23.2)	20 (29.0)	13 (18.8)	0.62
		Male	24 (33.3)	19 (26.4)	21 (29.2)	8 (11.1)	
		Total	44 (31.2)	35 (24.8)	41 (29.1)	21 (14.9)	
	2	Female	36 (44.4)	18 (22.2)	10 (12.3)	17 (21.0)	0.26
		Male	24 (29.6)	22 (27.2)	11 (13.6)	24 (29.6)	
		Total	60 (37.0)	40 (24.7)	21 (16.7)	41 (25.3)	
	3	Female	14 (28.0)	15 (30.0)	11 (22.0)	10 (20.0)	0.26
		Male	19 (37.3)	20 (39.2)	7 (13.7)	5 (9.8)	
		Total	33 (32.7)	35 (34.6)	18 (17.8)	15 (14.9)	
	4	Female	20 (37.7)	13 (24.5)	13 (24.5)	7 (13.2)	0.43
		Male	18 (34.0)	15 (28.3)	8 (15.1)	12 (22.6)	
		Total	38 (35.9)	28 (26.4)	21 (19.8)	19 (17.9)	
**U20**	1	Female	31 (32.3)	20 (20.8)	24 (25.0)	21 (21.9)	0.72
		Male	27 (27.0)	24 (24.0)	30 (30.0)	19 (19.0)	
		Total	58 (29.7)	44 (22.4)	54 (27.5)	40 (20.4)	
	2	Female	31 (30.4)	25 (24.5)	24 (23.5)	22 (21.6)	0.87
		Male	31 (30.1)	22 (21.4)	23 (22.3)	27 (26.2)	
		Total	62 (30.3)	47 (22.9)	47 (22.9)	49 (23.9)	
	3	Female	24 (35.8)	20 (29.9)	14 (20.9)	9 (13.4)	0.98
		Male	23 (34.3)	22 (32.8)	14 (20.9)	8 (11.9)	
		Total	47 (35.1)	42 (31.3)	28 (20.9)	17 (12.7)	
	4	Female	21 (30.4)	18 (26.1)	14 (20.3)	16 (23.2)	0.06
		Male	19 (28.4)	31 (46.3)	9 (13.4)	8 (11.9)	
		Total	40 (29.4)	49 (36.1)	23 (16.9)	24 (17.6)	
**Seniors**	1	Female	17 (20.2)	24 (28.6)	20 (23.8)	23 (27.4)	0.22
		Male	24 (25.0)	15 (15.6)	26 (27.1)	31 (32.3)	
		Total	41 (22.8)	39 (21.7)	46 (25.5)	54 (30.0)	
	2	Female	26 (26.0)	28 (28.0)	19 (19.0)	27 (27.0)	0.92
		Male	28 (25.5)	27 (24.5)	24 (21.8)	31 (28.2)	
		Total	54 (25.7)	55 (26.2)	43 (20.5)	58 (27.6)	
	3	Female	11 (16.9)	15 (23.1)	17 (26.2)	22 (33.8)	0.26
		Male	20 (27.0)	21 (28.4)	17 (23.0)	16 (21.6)	
		Total	31 (22.3)	36 (25.9)	34 (24.5)	38 (27.3)	
	4	Female	22 (31.9)	15 (21.7)	16 (23.2)	16 (23.2)	0.29
		Male	15 (19.0)	23 (29.1)	23 (29.1)	18 (22.8)	
		Total	37 (25.0)	38 (25.7)	39 (26.3)	34 (23.0)	

Within the U18 age group, differences in the RAE prevalence among athletes from different events were found (Table 2). A pairwise comparison revealed a statistically significant difference between Group 1 and Group 2 (*p* = 0.003). The difference reported may be due to a higher number of athletes from Q3 in Group 1 (*p* < 0.001; OR 2.75 1.52–4.94) and a lower number of athletes from Q4 (*p* < 0.001; 0.52 0.29–0.93).

Within the U20 age group, there were also differences in the prevalence of the RAE among athletes from different events (Table 2). A pairwise comparison revealed a statistically significant difference between Group 1 and Group 4 (*p* = 0.024) where Group 4 had significantly fewer ”late-born” athletes (*p* = 0.016; OR 0.55 (0.31–0.98)). Additionally, a statistically significant difference between Group 2 and Group 4 was found (*p* = 0.048) where Group 4 had significantly fewer ”late-born” track and field athletes (*p* = 0.025; OR 0.68 (0.4–1.18)). Differences between Group 2 and Group 3 (*p* = 0.043) showed that Group 3 had significantly fewer ”late-born” track and field athletes (*p* = 0.016; OR 0.46 (0.25–0.84)).

Among senior athletes, the prevalence of the RAE did not differ among particular events (Table 2).

## Discussion

This study found a high prevalence of the RAE among most successful track and field athletes at U18 and U20 age groups. Nevertheless, among most successful Senior track and field athletes, this effect disappeared, primarily due to a significant increase in the number of ”late-born” athletes. Other earlier studies had also demonstrated a widespread RAE among young track and field athletes, starting at the age of 9–10 years (Brazo-Savavera et al., 2017; Brustio et al., 2021; Gundersen et al., 2022; Hollings et al., 2014).

Similar patterns have been observed among other popular sports, namely, soccer and hockey. The most likely reason for the widespread prevalence of the RAE in highly competitive youth sport may be due to the advantage of the biologically mature athletes which is evident in ”early-born” athletes when competing against their ”late-born” peers.

Serum testosterone concentration is a significant factor determining strength, speed, endurance and bone density. In addition, it promotes competitive behavior and consequently provides competitive advantage (Eklund et al., 2017; Hirschberg, 2020). According to Senefeld et al. (2020) the mean testosterone concentration in girls aged 6–20 years increases from 0.08 nmol/L to 1.02 nmol/L with a plateau beginning at the age of 14 years, and in their male peers from 0.07 nmol/L to 17.9 nmol/L (*p* < 0.001) with a plateau beginning at the age of 17 years (Senefeld et al., 2020). Therefore, children who are chronologically older and probably more biologically mature may have higher testosterone levels, and thus a physical as well as a cognitive advantage. Although the RAE and the degree of biological maturity are independent constructs, it seems clear that chronologically older young athletes are more likely to have a higher maturity status.

Our findings for the first time demonstrate the absence of the RAE among the most successful track and field senior athletes. Studies involving athletes from other highly competitive sports (e.g., soccer, rugby, basketball and hockey) have shown an increase in the number of ”late-born” athletes at the senior professional level, but still fewer than ”early-born” athletes (Bezuglov et al., 2020; Lupo et al., 2019; Parent-Harvey et al., 2014).

In our study, more ”late-born” athletes among the most successful ones were found, but also those athletes outnumbered ”early-born” athletes in the Senior age group. The difference in the ”late-born” and ”early-born” athletes ratio may be due to the specifics of different sports. For example, in soccer and hockey, children often start training and competing regularly before the age of 8–10 years and children who have not participated in these sports prior to the age of 10–12 years are regarded as having limited opportunity to be selected for elite soccer and hockey youth organizations. Furthermore, given that almost all highly competitive youth soccer organisations describe an extremely pronounced RAE, ”late-born” athletes simply have no chance of getting into elite youth hockey and soccer. However, the situation is different in athletics. Participation in athletics usually commences before 10–12 years of age, and athletes with well-developed strength and speed may enter the sport before 18–20 years of age, when the influence of biological maturation on physical qualities is no longer significant. The available research suggests that only a small proportion of athletes who were successful as juniors remains successful as seniors and that nearly 70% of successful senior athletes do not reach the top 100 in the U18 and U20 age groups (Bezuglov et al., 2022). That is, most of athletes in the top 100 at the senior level either enter athletics from another sport at around 20 years of age or were competing in athletics, but not successfully at U18 and U20 age groups.

Therefore, the task of scientists and practitioners is to develop solutions for the RAE prevention in sports. A good example is going beyond age groups and using exact date of birth and chronological age (Rüeger et al., 2023). Biological maturity accounting for performing athletes is considered a substantial method (Malina et al., 2015). Another possible solution is limiting the number of major competitions during adolescence, which speeds up training of young athletes and may negatively affect their long-term development. Biobanding is a possible solution, as it has already shown its effectiveness in soccer. Biobanding can benefit all groups of athletes by providing fair competition and new challenges for both early and late maturing athletes (Lüdin et al., 2022; Malina et al., 2019).

Further research is warranted and should examine factors influencing the possible reasons for the prevalence of the RAE and measures to reduce the negative impact on the selection process in highly competitive youth sport.

## Conclusions

Among the most successful track and field athletes, the RAE was only pronounced in the juvenile years (U18 and U20 age groups). Among the most successful senior track and field athletes, the number of ”late-born” athletes exceeded the number of athletes born in all other quarters, including the first quarter, which is most likely due to the specific nature of the sport.
